# Case note and chart review of mortality in patients with a predicted low risk of death on admission to the ICU

**DOI:** 10.1186/cc9879

**Published:** 2011-03-11

**Authors:** S Elia, R Fisher, M Mostert

**Affiliations:** 1 University Hospital Lewisham, London, UK

## Introduction

The aim was to establish whether suboptimal care or system failures in the delivery of care contributed to mortality in patients admitted with a predicted low risk of death to our ICU.

## Methods

We defined low risk of death as a predicted mortality of less than 20% based on either the ICNARC or APACHE II risk prediction models [1]. We reviewed the case notes and ICU charts of patients with a low risk of death admitted to our ICU during July to December 2008 and April to September 2009.

## Results

Seven hundred and fifty patients (799 admissions, 49 readmissions) were admitted during the periods under review. The hospital mortality rate was 20.7% (155 patients) and of the 155 nonsurvivors 29 patients had a predicted low risk of death. Case notes for five patients could not be obtained and notes and charts for 24 of the 29 patients were reviewed. Errors identified in data collection: in two patients, incorrect data collection was identified that may have underestimated the risk of death. Suboptimal care identified: in four patients (16.7%), five instances of suboptimal care or system failures in care delivery were identified - delay in obtaining investigations (one laboratory, one radiology) delayed definitive treatment (two cases), delay in referring patient to the ICU (one case), elective surgical procedure caused bowel injury in a high-risk patient (one case), and delay in obtaining medical records caused the inappropriate admission of a patient to the ICU (one case). Patients with severe progressive illness: some patients were admitted with a low physiology score and low predicted risk of death but with a poor prognosis due to an underlying progressive illness.

## Conclusions

A case note review of ICU nonsurvivors can identify areas where service delivery and patient safety can be improved. Four patients (16.7%) with alcoholic liver disease (ALD) died despite a low physiology score on admission. The increased incidence of ALD in our unit is in line with the national trend.
